# Extensor Indicis Proprius Transfers Versus Tendon Graft in Extensor Pollicis Longus Reconstruction: A Systematic Review and Meta‐Analysis

**DOI:** 10.1111/ans.70558

**Published:** 2026-03-08

**Authors:** Daniel J. Keating, Simon J. Maciburko, Pradyumna Herle, Anthony C. Berger

**Affiliations:** ^1^ St Vincent's Hospital Melbourne Fitzroy Victoria Australia

## Abstract

**Background:**

In extensor pollicis longus ruptures not amenable to primary repair, secondary reconstruction of the tendon has traditionally been performed using either extensor indicis transposition or free tendon graft techniques. This paper constitutes a systematic review and meta‐analysis of the current literature with a focus on Geldmacher's scoring to identify the effectiveness of these procedures.

**Methods:**

We performed a literature search of medical databases to identify papers fulfilling the inclusion criteria. A meta‐analysis abiding by Preferred Reporting Items of Systematic Reviews and Meta‐analyses was completed with Geldmacher's score as a primary outcome and objective measurement of functional recovery following each procedure.

**Results:**

Nine papers were identified and included in the meta‐analysis following independent review by three researchers. On analysis of the data, “very good” and “good” Geldmacher's scores were achieved in 81.8% of patients who underwent extensor indicis transfer and 87.5% of patients who underwent tendon grafting. These results suggest that both techniques represent an equivalent return to postoperative function.

**Conclusions:**

This systematic review and meta‐analysis found that both extensor indicis transfer and tendon graft provide a practically equivalent return to function following extensor pollicis longus reconstruction. However, clinical limitations of tendon grafting, along with theoretical models of tendon repair may suggest extensor indicis as a more reliable means of reconstruction.

## 1 Introduction

Extensor pollicis longus (EPL) injury is a common clinical presentation for the hand surgeon and has the potential to result in significant morbidity for patients. Damage to the EPL tendon primarily results in loss of thumb retropulsion and reduced extension of the interphalangeal (IP) and metacarpophalangeal (MCP) joints [1, 2].

EPL tendon division can be classified into two distinct categories—open and closed injuries. Most open injuries, typically secondary to sharp, penetrating trauma, are commonly amenable to primary repair if treated acutely. Closed ruptures are often a result of chronic inflammation, stenosing tenovaginitis, tenosynovitis, steroid injections, or systemic steroid use and therefore are more commonly seen in patients with underlying rheumatoid conditions and arthropathies [3, 4]. Furthermore, spontaneous nontraumatic EPL ruptures can often be precipitated by attrition against abnormal bone ridges, osteophytes, or metalware following distal radius fractures [2, 3]. Under these circumstances, prolonged damage to the tendon and fraying of the rupture site may prevent direct repair. In these cases, further reconstruction with a tendon graft or tendon transfer may be necessary to restore function. While there are numerous methods of tendon reconstruction, the predominant techniques remain the extensor indicis (EI) transfer and the palmaris longus intercalated tendon graft.

While these methods have been described in detail in the literature, direct comparison in functional outcomes between the two methods has not been well established using validated scoring systems.

In this paper, our authors reviewed these two techniques through a systematic review and meta‐analysis utilizing the Geldmacher scoring system in order to identify any functional differences and discuss the relative merits of each method.

## 2 Methods

### 2.1 Research Ethics

PRISMA guidelines were adhered to in this meta‐analysis.

### 2.2 Search Strategy

An electronic literature review was completed using PubMed and Ovid Medline for publications dated from 1970 to 2025.

Searches in the literature were as follows:

1. Medline Searches
  - (MH “Tendon/TR”) AND (MH “Tendon Transfer”) AND (MH “Thumb”) *n* = 74
  - (MH “Tendon/TR”) AND (MH “Autograft”) AND (MH “Thumb”) *n* = 4
  - (MH “Tendon Injuries”) AND (MH “Tendon Transfer”) AND (MH “Thumb”) *n* = 107
  - (MH “Tendon Injuries”) AND (MH “Tendon Transfer”) AND (MH “Hand Strength”) *n* = 12
  - (MH “Tendon Transfer”) AND (MH “Thumb”) AND (MH “Hand Strength”) *n* = 37
2. Pubmed Searches
  - “Tendons/transplantation”[Mesh] AND “Thumb/injuries”[Mesh], *n* = 40
  - “Tendon transfer” [Mesh] AND “Thumb/injuries”[Mesh], *n* = 78
  - “Thumb”[MeSH Terms] AND “Tendons/surgery”[Mesh], *n* = 426

**FIGURE 1:**
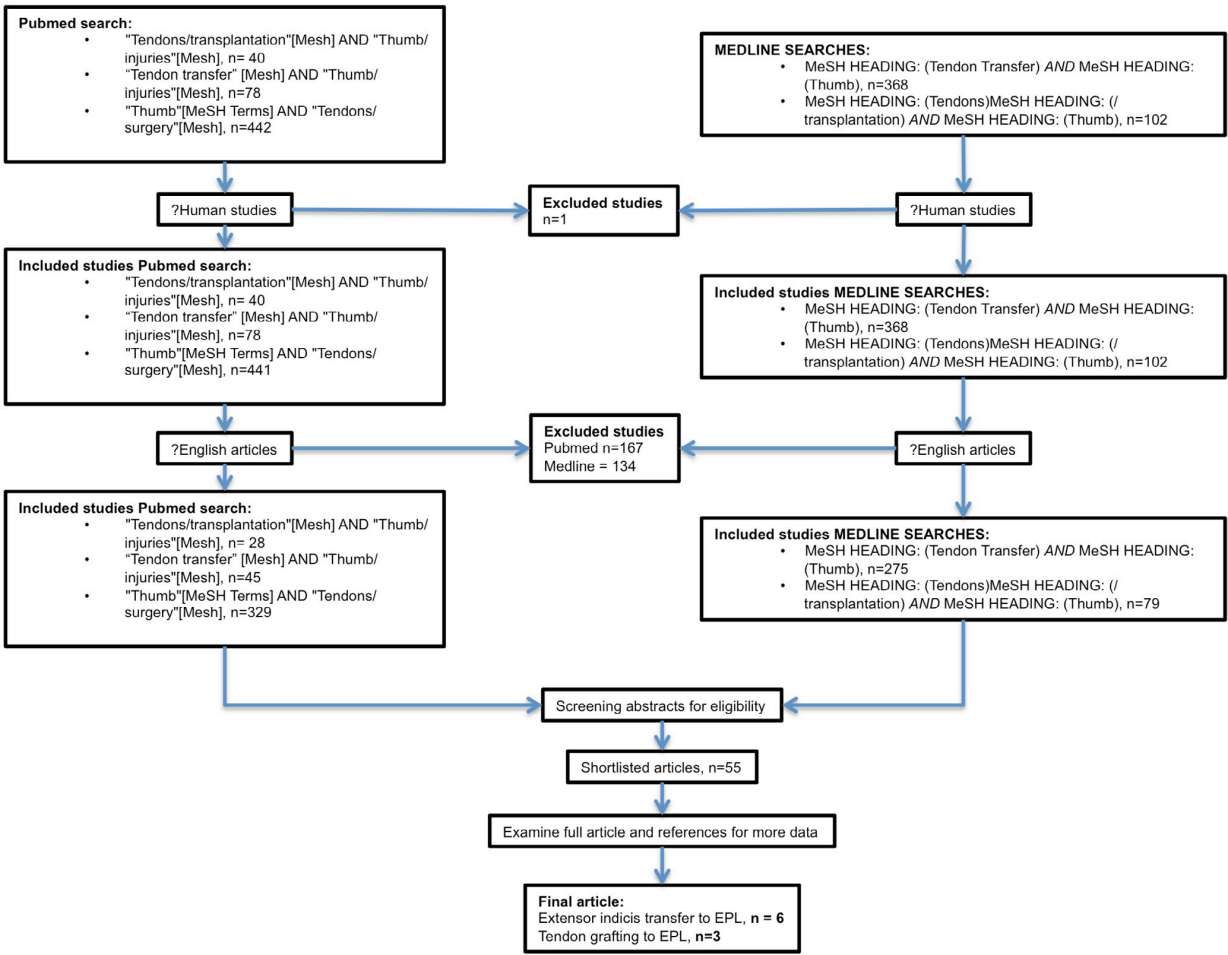
Search terms for meta‐analyses.

Article abstracts and/or full articles were then reviewed separately by three authors to determine the suitability of each article for inclusion. The inclusion criteria for final analysis were (i) explicit statement of numbers in EI tendon transfer and/or tendon grafting groups, AND (ii) outcomes defined in terms of the Geldmacher ratings of “very good,” “good,” “fair” or “poor” for each of the outcomes, AND (iii) statements of demographic details for patients enrolled in the study (gender, mechanism, and mean age) were provided. Papers that were selected for inclusion were also examined manually to find additional associated literature. Search items were excluded if they were not published in peer‐reviewed journals or if they were published in non‐English literature. Animal and cadaver studies were also excluded. Article selection is summarized in Figure 1. A list of papers that were initially shortlisted but later excluded was kept throughout this process. Authors with multiple papers regarding the topic had only their most recent publication included in the analysis.

Article abstracts and/or full article details were then reviewed by three separate reviewers (D.J.K., S.J.M., and P.H.) in order to determine article suitability. The criteria applied to article selection for the final inclusion for double limb analysis were (i) explicit statement of numbers in extensor indicis tendon transfer and tendon graft groups AND (ii) outcomes defined in terms of Geldmacher rating of excellent, good, fair, or poor for each of the outcomes AND (iii) explicit statements of demographic details for patients enrolled in the study in terms of gender, mechanism, and mean age. Papers included were also manually examined to find additional associated literature.

Search items were excluded if they were not published in peer‐reviewed journals and non‐English articles. Non‐human studies were also excluded.

Article section is summarized in Figure 1. A list of excluded shortlist papers was kept through this process. Authors with multiple papers regarding the topic were analyzed with consideration of the most recent publication.

### 2.3 Statistical Analysis

Statistical analysis was a random effects pooled rate of events for EI transfer and tendon grafting for EPL tendon reconstruction (also known as a single‐arm meta‐analysis). This was done with the Comprehensive Meta‐Analysis program version 3 (CMA v3) and verified with the meta‐prop command in STATA 12 [5].

Meta‐regression analyses were then performed with the CMA v3 program where data permitted.

### 2.4 Definitions

The Geldmacher assessment score (Table 1) was selected as the primary outcome of our study to provide an objective and universal outcomes assessment system. Results were then clustered into two distinct groups of very good/good and fair/poor Geldmacher scores using the scales of 24–17 and 16–0, respectively. This was done in order to increase the numbers in each category and create a direct comparison in analysis.

**TABLE 1:** Geldmacher system for scoring results of extensor tendon repair.

Function	Range	Score
Radial abduction range (between thumb and index finger)	> 70°	6 points
	51°–70°	4 points
	31°–50°	2 points
	9°–30°	0 points
Elevation deficit	0.0–1.0 cm	6 points
	1.1–2.0 cm	4 points
	2.1–3.0 cm	2 points
	> 3.0 cm	0 points
Opposition distance	0.0–2.5 cm	6 points
	2.5–4.0 cm	4 points
	4.1–6.0 cm	2 points
	> 6.0 cm	0 points
Flexion extension deficit (difference compared to contralateral hand)	0°–5°	6 points
	6°–30°	4 points
	31°–60°	2 points
	> 60°	0 points

In terms of meta‐regression analyses, the definition of an open laceration was conferred to any study detailing “penetrating, sharp or laceration trauma.” No studies included in this review specifically mentioned open fractures as a mechanism of injury, and therefore, all fractures detailed in the literature were assumed to be closed injuries. Patients included in the rheumatoid arthritis subgroup were defined as those with chronic, degenerative injury with a confirmed diagnosis of rheumatoid arthritis.

## 3 Results

Six studies for EI tendon transfer and three studies of tendon grafting for EPL tendon reconstruction were included. The key features of these studies are summarized in Tables 2 and 3.

**TABLE 2:** Tendon transfer for EPL defect reconstruction.

Study name	Study year	Number of patients	Geldmacher very good/good	Geldmacher fair/poor	Age (mean)	Gender (% male)	Open Laceration (%)	Closed Injury/wrist fracture (%)	Rheumatoid arthritis (%)
Figl	2011	25	24	1	54	52	0	100	0
Noorda	1994	22	9	13	52	41	45	55	4.5
Schaller	2007	28	24	4	NR	62	50	50	0
Magnussen	1991	21	19	2	51	43	14	57	24
Lemmen	1999	17	11	6	40	76	53	47	0
Schneider	1983	25	23	2	NR	NR	26	44	30

Abbreviation: NR, not recorded or not recorded separately for different groups in the study.

**TABLE 3:** Tendon grafting for EPL defect reconstruction.

Study name	Study year	Number of patients	Geldmacher very good/good	Geldmacher fair/poor	Age (mean)	Gender (% male)	Open laceration (%)	Closed injury/wrist fracture (%)	Rheumatoid arthritis (%)
Hamlin	1977	12	12	0	41	67	42	42	0
Schaller	2007	17	13	4	NR	NR	NR	NR	NR
Mannerfelt	1990	16	15	1	NR	Nr	NR	NR	NR

Abbreviation: NR, not recorded or not recorded separately for different groups in the study.

Six studies for EI tendon transfer and three studies of tendon grafting for EPL tendon reconstruction were included. The key features of these studies are summarized in Tables 2 and 3. The estimated rate of very good and good Geldmacher scores in patients treated using EI transfer in a pooled estimate from six studies was 81.8% (60.4%–93.0%) [1, 2, 5, 6, 7, 8]. The estimated rate of very good and good Geldmacher scores post tendon grafts in a pooled estimate from three studies was 87.5% (66.4%–96.1%) [8, 9, 10].

**FIGURE 2:**
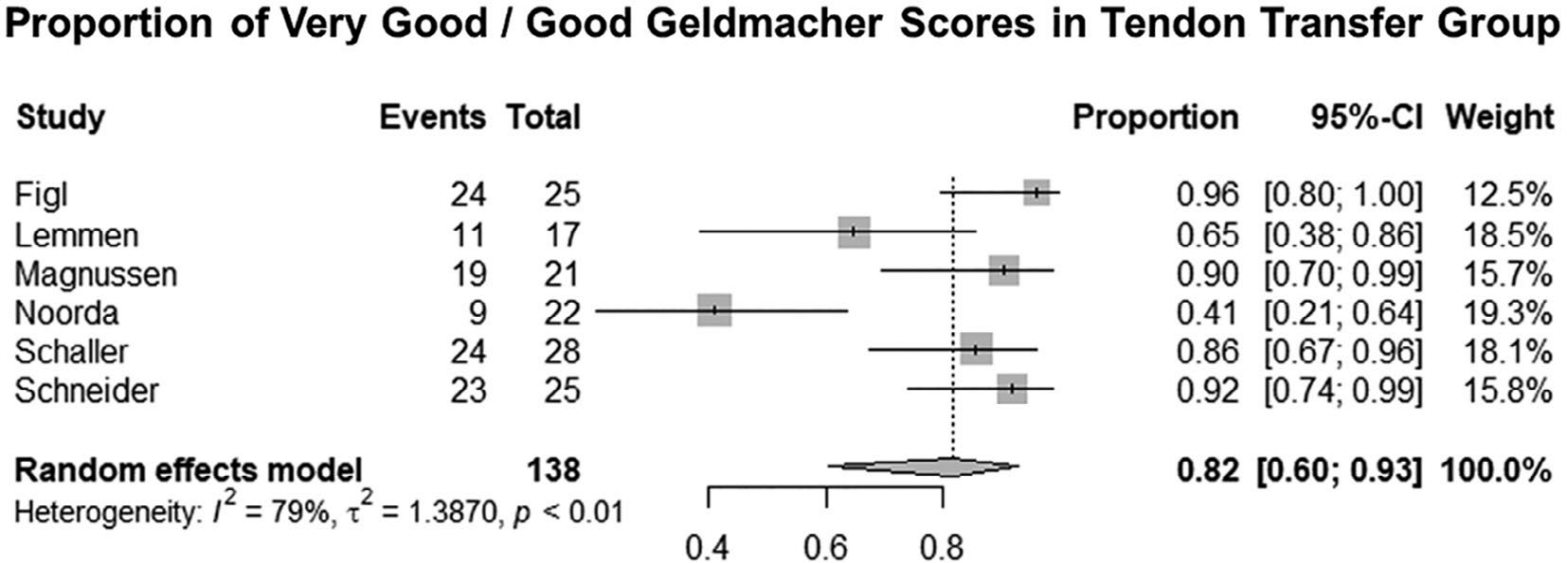
Pooled estimate rates, confidence interval, and heterogeneity testing of Geldmacher scores in EIP transfer.

**FIGURE 3:**
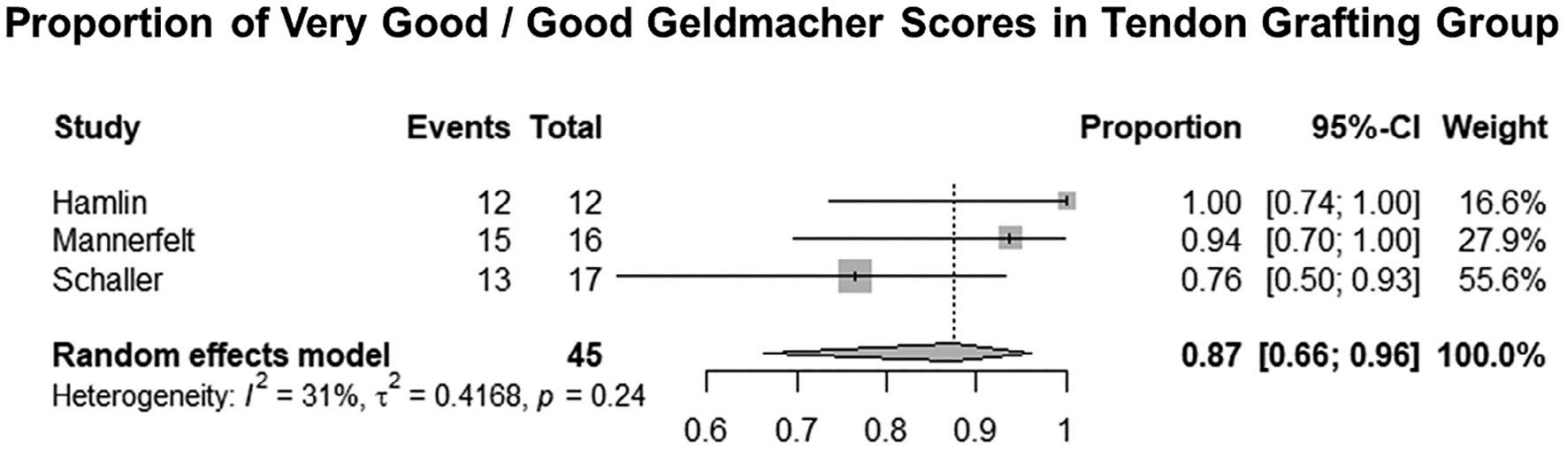
Pooled estimate rates, confidence interval, and heterogeneity testing of Geldmacher scores in tendon grafting.

The pooled estimate rates, confidence interval, and heterogeneity testing are summarized in Figures 2 and 3. Meta‐regression of age, percentage of patients with rheumatoid arthritis, and of factors pertaining to the mechanism of injury was performed in the EI transfer group. The results of meta‐regression analyses are summarized in Table 4 below.

**TABLE 4:** Meta‐regression of age and mechanism of injury.

Variable	Coefficient	*p*
Age	0.093	0.605
% Rheumatoid arthritis	0.0474	0.534
% Open lacerations	−0.0622	**0.0024**

*Note*: The bold is designed to emphasise the analysis/*p*‐value fulfilling the criteria for achieving statistical significance (*p* < 0.05).

Data for co‐variables that affect thumb function post tendon grafting was limited and this prevented us from performing a meaningful meta‐regression analysis for this technique.

## 4 Discussion

EPL tendon rupture can occur following open lacerations, closed distal radius fractures, or as a result of tenosynovitis secondary to rheumatoid arthritis [3, 4]. While acute penetrating lacerations are comparatively easy to treat, closed injuries and delayed presentations often require a more complex reconstruction. In delayed injuries, the loss of tendon length and muscle contracture necessitates the recruitment of extra tendon length via a transfer or graft [1]. Furthermore, fraying of the tendon in these injuries decreases the strength of the repair.

EPL tendon injury can occur after open lacerations or closed injuries. While acute penetrating lacerations are comparatively easy to treat, often necessitating direct repair, closed injuries and delayed presentations often require a more complex reconstruction.

Closed tendon ruptures are often the result of attrition from distal radial fractures, stenosis of the extensor compartments, or synovitis of the tendon sheaths as classically occurs in rheumatoid arthritis [3, 4]. In these cases, the loss of tendon length and muscle contracture necessitates. The recruitment of extra tendon length via a transfer or graft. Furthermore, the presence of an unsuitable tendon bed and frayed tendon edges increases the risk of recurrence if directly repaired.

The functional deficit following EPL rupture manifests mainly in the ability of the thumb to be extended in the plane of the palm and extension at the MCP joint and IP joint of the thumb, although it is important to note that a significant number of patients do not have a sufficient functional loss to warrant any reconstruction [1].

To characterize the severity of the functional deficit and to characterize the success of tendon repair functional scores have been created. The most popular of these is the Geldmacher score, a system that aggregates abduction, resting position, opposition, and general range of motion of IP and MCP joints of the thumb [8, 11]. Other modified scoring schemes have been used, including the DASH and quickDASH scores, but we have not included these articles in our review as there were too few of these articles to provide a statistically significant contribution.

The two main methods described for reconstruction of EPL tendon defects are free tendon grafting and tendon transfer [1, 8, 9, 10]. The most common tendon harvested for tendon grafting is the palmaris longus tendon, while the most common for transfer is the EIP tendon. Other options described for tendon transfer include abductor pollicis longus and extensor carpi radialis longus [12, 13, 14].

Our meta‐analysis demonstrates that the success of producing good and very good Geldmacher's scores was 81.8% for EI transfer and 87.5% for free tendon grafting. These results are comparable and suggest roughly equivalent success of both methods for thumb function reconstruction following EPL injury. While free tendon grafting did produce marginally higher results of very good/good Geldmacher's scores, the difference is not adequate to demonstrate clearly a more effective surgical technique.

In the absence of an objectively superior technique, several important questions are now raised. One, is there a way of selecting a demographic of patients that would be most appropriate for either procedure? Two, is there a way to predict which patients may have better or worse functional outcomes when either technique is used? And three, which technique has less donor site morbidity?

The first question cannot be addressed using a systematic review of the current data on the topic. Better‐designed prospective studies, preferably randomized controlled trials, which examine the outcomes of tendon grafting versus EI tendon transfer in EPL rupture, are clearly required.

We attempted to answer the second question by performing meta‐regression analyses to identify other factors that affect functional outcome, such as the variables of age, mechanism of injury, and number of patients with rheumatoid arthritis. This data would allow surgeons to select a subpopulation that require closer follow‐up or more intensive postoperative interventions. Meta‐regression analysis was unable to be performed on the limited data in the palmaris longus tendon grafting group. However, in the EI transfer group, meta‐regression analysis demonstrated that studies with higher proportions of open lacerations correlated negatively with very good/good Geldmacher or equivalent functional scores post EI transfer. While this association is statistically significant, the reasons behind this correlation are unclear. A potential reason could be that open lacerations may involve other surrounding neurovascular structures and other musculotendinous units when compared to closed trauma or non‐traumatic rupture.

A significant issue raised by both techniques of repair is the creation of a site for which the transfer/graft is harvested. The EI tendon lies in the fourth wrist extensor tendon compartment permitting harvest through the same incision used for EPL exploration. In EI transfers, the principal concern in sacrificing the tendon is a subsequent weakness in extension strength of the donor index finger. However, multiple studies have stated that this donor defect is of minimal functional importance [1, 7]. Furthermore, independent index extension may be preserved by sectioning the index tendon proximal to the dorsal extensor hood intraoperatively [15]. Despite this, individuals who require fine dexterity in the index finger in whom this donor deficit may not be acceptable [8]. As the degree of functional loss in the donor site has not been quantified in all reports, an overall estimate of the functional deficit created by EI tendon harvest is currently not possible.

In contrast to the access required for tendon transfer, tendon grafts require a separate incision to access the tendon. Some complications that have been previously reported include iatrogenic damage to the median nerve, the palmar cutaneous branch of the median nerve, adjacent tendons, and wrist flexion contractures [16]. The rate of these complications in patients undergoing EPL reconstruction is once again unclear. Furthermore, there are patients in whom the risks of additional incisions should not be minimized, such as those with a history of wound healing difficulty or a history of keloid or hypertrophic scarring.

Anatomical variation of both palmaris longus and EI tendons should also be considered when discussing donor site morbidity. Palmaris longus was found to be absent unilaterally in 16% and bilaterally in 9% of Caucasian patients, and 16% and 3.3% of the Indian population, respectively [17, 18].

In this patient population with an absent palmaris longus tendon, there is the additional morbidity and difficulty in seeking an alternative donor tendon site, including the opposite arm or the lower limb for the plantaris tendon. Conversely, the EI tendon is only very rarely absent, with few cases in the literature and an incidence rate of between 0% and 5% [19].

In comparing EI transfer and tendon grafting, the physiological models of tendon healing and neovascularization must also be considered on a theoretical level. Tendon grafts, due to their avascular nature, require a predominantly extrinsic healing mechanism at two separate junctions. Any intrinsic healing that occurs would do so in a retrograde fashion, increasing the risk of graft necrosis and technical failure or secondary rupture [7]. Furthermore, a reliance on extrinsic healing has been shown to result in greater adhesion formation and disruption to tendon glide, compromising functional return and rehabilitation [20].

Additionally, grafts require the motor unit of the EPL to remain functional. Therefore, extensive scarring and contracture of the EPL muscle precludes the use of tendon grafts. This is particularly so in rheumatoid arthritis patients, where tendon grafts have been shown to correct extensor lag, but at the cost of digital flexion due to reduced musculotendinous excursion [21].

EI tendon transfer is therefore the theoretically superior technique for EPL tendon reconstruction, due to minimal donor site morbidity, consistent anatomy, a predominantly intrinsic tendon healing mechanism and the ability to transfer a functional extensor unit for reconstruction. However, these theoretical benefits of EI transfer are not quantified in enough studies to make any statistically meaningful conclusions.

Aside from the surgical technique itself, the other factor that needs to be considered is variability in postoperative rehabilitation between the different studies. There is likely to be variability in access to, compliance, and exact exercises and regimens used in the rehabilitation period. A systematic review by Wood et al. suggests dynamic splinting confers better functional outcomes compared with static splinting following EPL repair [22]. However, no conclusions were drawn about early active movement, and further investigation is required. During our examination of the literature, there was no definitive evidence that dynamic splinting and early active motion groups had any significant differences in functional outcomes [23]. We believe that examination of early active movement and its effects on long‐term function in high‐risk groups is potentially a topic worth examining in future studies.

The limitations of this review include inherent heterogeneity of studies, their retrospective nature, and small numbers in included studies, all of which may skew a truly representative estimate of treatment effect. We have attempted to counter these by utilizing random effects modeling, using papers that reported at least 10 patients in each group and formally testing for publication bias.

## 5 Conclusions

The estimated good/excellent functional outcomes following EI tendon transfer or tendon grafting for use in EPL rupture are equivalent (81.8% and 87.5%, respectively). Our data suggest that patients with open lacerations are at a higher risk of poorer overall functional outcomes following EPL reconstruction with EI transfer and that more intensive follow‐up and more extensive postoperative hand therapy regimens are warranted in this group. Further investigation is needed in the form of randomized controlled trials comparing grafting and tendon transfer for definitive recommendations. The role of early active mobilization particularly for open lacerations in EPL, also warrants further investigation.

Donor site morbidity, while not adequately addressed in the articles, should come into consideration when deciding upon appropriate technique. Individuals with an absence of palmaris longus in the ipsilateral hand should be carefully considered for a tendon transfer as to minimize the morbidity of operating on more than one limb.

In the absence of contrary data, the authors suggest that functional outcomes with either repair are equivalent. EI transfer demonstrates several favorable anecdotal and theoretical advantages when compared to tendon grafting.

## Author Contributions

**Daniel J. Keating:** writing, editing, and data collection. **Simon J. Maciburko:** data collection and editing. **Pradyumna Herle:** statistical analysis. **Anthony C. Berger:** reviewing and editing.

## Funding

The authors have nothing to report.

## Ethics Statement

The authors have nothing to report.

## Consent

Informed consent was obtained from all individual participants included in the study.

## Conflicts of Interest

The authors declare no conflicts of interest.

## Data Availability

The data that support the findings of this study are available from the corresponding author upon reasonable request.
